# Vaginal microbioma and the presence of *Lactobacillus* spp. as interferences in female fertility: A review system

**DOI:** 10.5935/1518-0557.20230006

**Published:** 2023

**Authors:** Sabrina Vieira de Souza, Paula Bruno Monteiro, Gabriel Acacio de Moura, Nayara Oliveira Santos, Cristina Tonin Beneli Fontanezi, Isadora de Almeida Gomes, Clara Andrade Teixeira

**Affiliations:** 1 Graduating in biomedicine at the Christus University Center - UNICHRISTUS, Fortaleza, CE, Brazil; 2 Master in Public Health from the Federal University of Ceará - UFC, Fortaleza, CE, Brazil; 3 Master in Veterinary Sciences from the State University of Ceará - UECE, Fortaleza, CE, Brazil; 4 Master in Medical Microbiology from the Federal University of Ceará - UFC, Fortaleza, CE, Brazil; 5 PhD in Experimental Pathology from the Faculty of Medicine of Ribeirão Preto da University of São Paulo (FMRP/USP), Ribeirão Preto, SP, Brazil

**Keywords:** microbiome, Lactobacillus, infertility, reproduction, dysbiosis

## Abstract

The vaginal microbiome is dominated by *Lactobacillus* spp. and the depletion of these microorganisms have been associated with adverse conditions that can affect women’s health. Disturbance of the vaginal niche with a non-lactobacillary microbiota is associated with susceptibility to some diseases, such as obstetric alterations and infertility, resulting in failure in natural pregnancies and increased demand for assisted reproduction treatments. The present study sought to understand the influence of *Lactobacillus* spp. and fertility female. A systematic search was performed in the following databases: PubMed, MEDLINE, SciELO and LILACS, using the keywords: “Microbiome”; “*Lactobacillus*” and “Female Infertility”, published in the last five years. The search resulted in 92 articles; however, 38 articles were excluded due to duplicity, 23 articles were excluded in the selection title/abstract, leaving 31 articles for full reading. In the end, 18 articles were analyzed. The studies encompassed a total of 2,011 women, using 27 types of samples to verify the composition of the microbiome. The eighteen articles that reported the microbiome of fertile women were constituted by a dominance of *Lactobacillus* spp. who joined to positive predictive outcomes in reproduction, while infertile women showed a dysbiotic profile. Therefore, analyzing bacterial patterns would allow a personalized diagnosis, which could favor personalized therapy for prevention and treatment of certain diseases.

## INTRODUCTION

The microbiota is the formation of communities of microorganisms that live inside or on the external surface of the human body and its genomic constitution is called microbiome. Under ideal circumstances, the vaginal microbiota is populated by over 200 bacterial species, which suffer genetic, environmental and behavioral influences, in addition to be influenced by the oral, rectal and penile microbiota (Auriemma *et al*., 2021; Martin, 2012; Mendling, 2016).

The healthy vaginal microbiota consists mainly of resident species of Lactobacillus, such as *L. crispatus, L. iners, L. jensenii* and *L. gasseri*. These microorganisms act as probiotics and inhibit the overgrowth of other bacterial species, for several direct and indirect antipathogenic mechanisms. Directly by producing active components such as lactic acid and hydrogen peroxide (H_2_O_2_), which kill or directly inhibit pathogens. In an indirect way, they form microcolonies that adhere to the epithelial cells and create a physical barrier against the adhesion of certain microorganisms, in addition to promoting the stimulation of host defense mechanisms against infections Sexually Transmitted Diseases (STIs) (Auriemma *et al*., 2021; Ceccarani *et al*., 2019; Franasiak & Scott, 2015; Jespers *et al*., 2012; Younes *et al*., 2018).

Throughout the menstrual cycle, hormonal fluctuations influence conditions environmental conditions of the vaginal lumen and, in turn, resident bacteria. The vaginal microbiota of mother serves as a source of colonization for the baby and around the first two to four weeks after birth, maternal estrogen promotes proliferation and thickening of the vaginal mucosa. Subsequently, the accumulation of lactic acid leads to a decrease in pH vaginal. This phase is short-lived, as maternal estrogen is metabolized, the epithelium vagina begins to thin and the glycogen levels decrease, thus raising the pH vaginal. In prepuberty, the microbiota is populated by a wide range of species of aerobic, strictly anaerobic and enteric bacteria, being compared to that of women adults with bacterial vaginosis. With menarche comes follicular development, leading to systemic production of estrogen, which causes the vaginal epithelium to begin to thicken and increase deposition of glycogen, mainly in the intermediate cells. The epithelial maturation ends up selecting microorganisms such as *Lactobacillus, Atopobium, Leptotrichia, Leuconostoc, Megasphaera, Pediococcus, Streptococcus* and *Weissella*. The fluctuations hormones throughout the menstrual cycle influence environmental conditions and transform inhabiting bacteria. Seen therefore an increase in the rate of *Lactobacillus* throughout the cycle menstrual cycle and, in contrast, the concentration of non-*Lactobacillus* species tend to be higher in menstruation. In post-menopause there are low concentrations of *Lactobacillus* and other bacteria, allowing the growth of a variety of other pathogenic species and enteric. As the epithelium becomes very thin as estrogen levels decrease, reduces the production and secretion of glycogen (Godha *et al*., 2018).

We understand, therefore, that the vaginal microbiota is mainly dominated by *Lactobacillus spp*. and depletion of these organisms is associated with several adverse conditions such as premature birth, pelvic inflammatory disease, increased risk of STIs such as Human Immunodeficiency Virus (HIV), Herpes Virus (HSV), Papillomavirus Human (HPV), chlamydia, trichomonas and multiple symptoms affecting quality of life female (Buchta, 2018; van Oostrum *et al*., 2013; Younes *et al*., 2018).

Clinically, the disturbance of the vaginal niche, with a non-lactobacillary microbiota, characterized as dysbiosis. Dysbiosis is defined as the imbalance of populations and/or microbiota functions and changes in microbiome diversity, being associated with the mostdifferent sites in the human body. In certain sites, dysbiosis can promote disease inflammatory bowel diseases, metabolic disorders, multiple sclerosis, allergies, asthma, autism and cancer (Requena & Velasco, 2021; Weiss & Hennet, 2017).

Recently, the imbalance of the vaginal microbiota has been pointed out as a possible interfere with female fertility. The World Health Organization (2020) classifies the infertility as the inability of a couple of reproductive age to conceive within a period of 12 months having sex without the use of contraceptives. Infertility can be caused by a series of factors: sexual diseases, obesity, smoking, sedentary lifestyle, illicit drugs, alcoholism, exposure to chemicals, radiation, stress, activities physical excess, non-recommended diets and age.

It is important to emphasize that the causes of infertility can be found in women or men, there are also the joint causes of male and females for couple infertility. Regarding infertile women, they have already been predisposing factors such as endometriosis, ovulatory problems, and age group (Félis *et al*., 2019; Starc *et al*., 2019).

Since the factors underlying infertility are complex and wide-ranging, approximately 40% of cases cannot be explained by anovulation or pathology tubal; these cases are defined as ‘unexplained infertility’ or ‘female infertility unspecified’. The “unexplained infertility” is much discussed since the diagnosis can be related to lack of a specific test, due to misdiagnosis or factors psychological (Félis *et al*., 2019; Hong *et al*., 2020).

With the development of state-of-the-art sequencing technology, high yield, the function of many bacteria considered normal in the vagina has been redefined. They developed concern not only about potentially pathogenic, but also in terms of changes in the entire structure of the vaginal microbiota. New molecular technologies may shed light on the role of bacteria in health gynecology, and also to elucidate how the change in the vaginal microbiota affects the susceptibility to diseases (Félis *et al*., 2019; Oliveira *et al*., 2019).

Female infertility brings serious psychosocial consequences, therefore, the prevention and management of female infertility are an integral component of services comprehensive sexual and reproductive health. Assisted reproduction has become an element comprehensive care for many women who have suffered from infertility over the past forty years (Esteves *et al*., 2019).

Assisted reproduction are the techniques used in the treatment of infertility, which manipulation of one or both gametes will take place. There are numerous techniques such as: intrauterine insemination (IUI), in vitro fertilization (IVF), intracytoplasmic injection of sperm (ICIS) (Souza & Alves, 2016).

In 2019, the Latin American Network of Assisted Reproduction announced that Brazil led the Latin American ranking of countries that performed the most breeding techniques assisted: 44,705 IVF cycles. In 25 years, 83,000 Brazilian babies were born through assisted reproduction treatments, demonstrating that assisted reproduction techniques have more evidence gained, since the decrease in fertility is an inevitable biological factor, combined with late motherhood (Foizer *et al*., 2014; Zegers-Hochschild *et al*., 2020).

Human reproduction can be considered inefficient, since the conception rate is 25-30% per cycle, of these, only 50% will pass by the 20^th^ week of pregnancy; and of gestational losses, 75% are the result of implantation failure that are not recognized by the clinical point of view. About 5% of women will have at least two consecutive losses, while 75% will have at least one implantation failure (Borges Júnior *et al*., 2020).

With the growth of assisted reproduction techniques, it has been studied even more on the interference in their success. Some studies correlate that pathogens such as *Mycoplasma tuberculosis, Chlamydia trachomatis* and *Neisseria gonorrhoeae*. When present in the vaginal microbiome interfere with fertility and reproductive techniques assisted (Sirota *et al*., 2014).

Since the literature has discussed that some microorganisms when present in the microbiome of infertile women, can disrupt or decrease implantation rates in assisted reproduction treatments, this study was designed to establish which influence of *Lactobacillus spp*. in female fertility.

## MATERIAL AND METHODS

This is a systematic literature review that addresses the influence of *Lactobacillus spp*. in female fertility. Studies that contributed to the hypothesis raised and, thus, to understand, scientifically, the relationship of changes in *Lactobacillus spp*. in the vaginal microbiota and in female fertility, highlighting the level of reliability and its clinical potential. It is noteworthy that it did not involve interventions in humans, therefore approval by an Ethics Committee was not required. We use the quote: “Preferred Reporting Items for Systematic Reviews and Meta-analysis” (PRISM) to report the results.

### Search strategy

This systematic literature review started in August 2021 until April 2022. The following databases were used: PubMed, MEDLINE, SciELO and LILACS, using the keywords: “Microbiome”; “Lactobacillus” and “Female Infertility”, in English and Portuguese, in advanced search. Articles published in the last five years were used (from 2017 to April 2022) in order to provide the most up-to-date and recent data.

### Article selection criteria

Full, original articles, in English, addressing infertility were included. female, microbiome, vaginal microbiota and assisted reproduction techniques. Were excluded, articles dealing with male infertility, review articles, case reports, animal studies, incomplete or unavailable.

### Measurements

Articles were analyzed based on inclusion and exclusion criteria. After this choice, a complete reading was performed, where it was possible to identify the contribution to this job. The following were observed: the studied group (healthy women and infertile women), the assisted reproduction techniques portrayed in the study, the methodologies used in the studies, the main microorganisms correlated with the lactobaciliary change, the of success and failure in the use of assisted reproduction techniques in women with dysbiotic profile and other factors underlying changes in the vaginal microbiome.

## RESULTS

### Data collection and analysis

**Figure 1:**
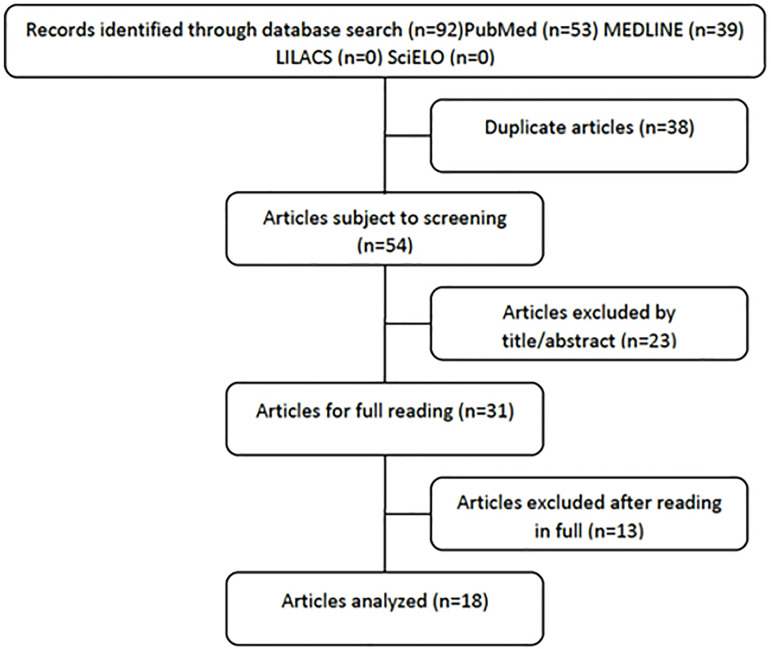
Articles analyzed.

The records identified in the data search were: PubMed (n=53), MEDLINE (n=39), SciELO (n=0), LILACS (n=0), resulting in a total of 92 articles. after the search initial period, the duplicate articles were removed, which were 38 articles, leaving, therefore, 54 articles for screening. After reading the title and abstract, 23 articles that did not met the inclusion criteria, leaving 31 articles for full reading. After full reading, 18 articles were analyzed (Figure 1).

### Description of included studies

The articles analyzed in the present study were published between 2017 and 2022 and met the inclusion and exclusion criteria. The studies encompassed a total of 2,011 women, being 512 (25.45%) fertile women and 1,499 (74.54%) infertile women.

Among the methodologies used in studies to analyze the vaginal microbiome: 14 (77.77%) of the studies used amplification of the 16S ribosomal RNA (rRNA) gene, 2 (11.11%) studies used quantitative real-time PCR (Wee *et al.,* 2018; Haahr *et al*., 2019) associated the use of these techniques, 1 (5.55%) study used culture aerobic, anaerobic and fungal routine, however Graspeuntner *et al*. (2018) and Azpiroz *et al.* (2021) used the three methodologies, 1 (5.55%) used the IS-pro technique. Graspeuntner *et al*. (2018) used complementary techniques, such as: ELISA and Immunblot.

The studies used 27 types of samples to verify the composition of the microbiome were: 14 (51.85%) vaginal swabs, 6 (22.22%) endometrial fluids and endometrial tissue, 3 (11.11%) cervical swabs, 1 (3.70%) rectal swabs, 1 (3.70%) urinary sample, 1 (3.70%) fecal sample, 1 (3.70%) cervix.

As for the association with assisted reproduction techniques: 12 (66.66%) studies addressed IVF, and Bernabeu *et al*. (2019), Koedooder *et al*. (2019) and Patel *et al.* (2022), jointly address ICSI, 1 (5.55%) study addressed patients submitted to assisted reproduction technologies without specifying the technique and 5 (27.77%) studies did not make the association between microbiota and assisted reproduction technique (Table 1).

**Table 1:** Studies.

Study	Group of Study	Age	Methodology used	Techniques assisted by reproduction	Microbiome/ microbiota
**Babu *et al*., 2017**	200 women (84 healthy - Group and 116 infertile - group 2)	18 years to 45 years	All swabs were subjected to culture aerobics, anaerobic and fungal from routine.	Does not portray	Group 1 - was dominated by *Lactobacillus* followed by *Micrococcus, Enterococci* and *Staphylococcus* spp. negative coagulase. Group 2 - *Candida spp., Enterococcus* followed by bacilli Gram negative, like *Escherichia coli*.
**Wee *et al*., 2018**	31 women (16 healthy 15 infertile)	28 years to 49 years	Collection of samples vaginal, cervical and endometrial analyzed by amplification of RNA gene ribosomal 16S (rRNA) and expression endometrial from human genes selected by the reaction in chain of polymerase from transcription reverse quantitative.	Does not portray	It was observed a dominated microbiota By *Lactobacillus*. However, there were a trend of that women infertile had more often *Ureaplasma* in vagina and *Gardnerella* in the cervix.
**Graspeuntner *et al*., 2018**	210 women (26 women infertile, 21 women by having infertility infectious, 89 women fertile, 54 professionals of sex.	Does not portray	Were used swabs for culture bacterial conventional, swabs for PCR against *C. trachomatis, N. gonorrhoeae, M. genitalium, M. hominis* and *U. urealitic*, swabs for the sequencing of the 16S gene rRNA.	Does not portray	It was observed *Lactobacillus* as the most gender prominent, which declined among the women's groups infertile and professionals of sex. In contrast had an increase in genus *Gardnerella* and *Prevotella* us women's groups infertile and professionals of sex.
**Bernabeu *et al*., 2019**	31 women (in treatment of reproduction assisted)	18 years to 50 years	Samples vaginal were harvested for analyze the V3 V4 region of the 16S rRNA and so if analyze the microbiome vaginal	Fertilization in in vitro (IVF) and Stimulation Ovarian controlled and injection intracytoplasmic the of sperm (ICSI).	Points a dominated microbiota by *Lactobacillus* (<90% Lactobacillus *spp*.) and one does not dominated by *Lactobacillus*, where the proportion of bacteria of the genus Gardnerella exceeds 10%.
**Haahr *et al*., 2019**	120 women (submitted to fertilization in vitro)	Age average of 40 years (Detour pattern 4.3)	Samples vaginal sequenced using the region V4 of the gene RNA ribosomal 16S with grouping of clades genomics of Gardnerella vaginalis.	Fertilization *in vitro* (IVF)	It was observed *Lactobacillus* as the dominant gender, however, the highest scores of Nugent were associated with greater diversity and included highs OTU loads corresponding to G. vaginalis, *A. vaginae, Sneathia* bloods and *Prevotella spp*.
**Koedooder *et al*., 2019**	192 women (67 women What got pregnant and 125 that don't got pregnant)	20 years to 44 years	The composition of the microbiota vaginal was determined using the IS-pro technique.	Fertilization *in vitro* (IVF) and Stimulation ovarian controlled and injection intracytoplasmic the of sperm (ICSI).	Dominance of *Lactobacillus crispatus* was an important factor in prediction of pregnancy.
**Fu *et al*., 2020**	67 women (27 failed applicant of implantation and 40 group control)	33.4±3.7	Sequencing the 16S gene rRNA of microbiota vaginal	Fertilization *in vitro* (IVF)	It was observed a dominance of *Lactobacillus* in control group, in addition of that it was verified that the decrease of *Lactobacillus*, plays an important role in pathogenesis of failure applicant of implantation.
**Zhao *et al*., 2020**	122 women (30 women infertile and 92 women healthy)	23 years to 40 years	Analysis of composition of microbiome vaginal using sequencing of the 16S gene rRNA.	Fertilization *in vitro* (IVF)	Infertile women present a decrease significant in diversity and richness of the microbiome in comparison with healthy women during the period of no ovulation (phase follicular), while the microbiome
**Carosso *et al*., 2020**	15 women (submitted to techniques of reproduction assisted)	Age ≤ 42 years old	A swab vaginal and the far end tip distal of the ET catheter were analyzed using the sequencing of the gene 16 SrRNA of last generation.	Fertilization *in vitro* (IVF)	The relative proportion of Lactobacillus vaginal and endometrial was decreased during the fertilization cycle *in vitro*, with a simultaneous increase of bacteria potentially pathogenic, such as *Atopobium Escherichia-Shigella* and *Prevotella*.
**Karaer *et al*., 2021**	223 women	23 years to 39 years	The samples vaginal for identification of microbiota vaginal was carried out using sequencing last generation and categorized from according to region hypervariable V3-V4 in gene region 16S rRNA.	Fertilization *in vitro* (IVF)	presented dominance of *Lactobacillus* in vaginal microbiota. the abundance relative of Streptococcus and *Gardnerella* was increased in women who don’t got pregnant.
**Hao *et al*., 2021**	100 women (51 subjected to IVF, where 25 got pregnant, 49 submitted transfers of embryo, where 27 They were clinically pregnant)	20 years to 40 years old age	The regions variables 3 and 4 (V3-V4) of 16S rRNA gene were amplified and sequenced.	Fertilization *in vitro* (IVF)	a minor proportion of *Lactobacillus* for other bacteria in cervical microbiota was associated with decrease in chances of pregnancy clinic.
**Sun *et al*., 2021**	184 women	Age Reproductive	Samples vaginas, from cervix and of the cavity uterine for sequencing of 16S rRNA.	Does not portray	It was observed that the microbiota cervico vaginal was dominated by *Lactobacillus*. At the general, the composition of the microbiota in uterine cavity was more diverse than that in the vagina and cervix of the uterus.
**Koedooder *et al*., 2021**	85 women (in treatment of reproduction assisted)	18 years to 43 years old	The composition microbial was Determined by the reaction in chain of polymerase of V1-V3 regions of the gene bacterial 16S rRNA	Fertilization *in vitro* (IVF)	A decrease significant in plenty of species of *Lactobacillus* as well like an increase significant in the species of *Staphylococcus* was observed in women who got pregnant after treatment of IVF/IVF-ICSI.
**Azpiroz *et al*., 2021**	307 women (287 infertile and 20 group control)	21 years to 39 years old.	Samples of swab were collected from vagina and the straight. The composition microbial by NGS and the expression of miRNA by PCR in time real.	Fertilization *in vitro* (IVF)	Infertile patients showed lower bacterial richness and increase in ratio Firmicutes / Bacteroidetes in rectal level and augmentation of reason *Lactobacillusbrevis* / *Lactobacillusiners* in samples vaginal in relation to the fertile group.
**Lüll *et al*., 2022**	25 women (with infertility)	28 years to 42 years old	Analysis of sample of fabrics and fluids for sequencing of 16S rRNA.	Fertilization *in vitro* (IVF)	It was observed that the dominance of *Lactobacillus* genus is a factor important that influences the composition microbial.
**Sezer *et al*., 2022**	52 women (26 infertile and 26 fertile)	20 years to 45 years old	Samples vaginal and endometrial were analyzed by PCR quantitative in real time.	Does not portray	It was found that the microbiota in vaginal samples from patients with infertility, it was harmed as much to the number of *Lactobacillus*. In addition addition, it was portrayed that the women with microbiota lactobaciliary impaired had increased risk for infertility.
**Villani *et al.*, 2022**	90 women (with diagnosis in infertility, 2 patients excluded, remaining 88, which 39 got pregnant and 49 negative)	24 years to 40 years old	Vaginal swabs which after the extraction of DNA microbial, the regions variables V3-V4 of the gene 16S rRNA were amplified and sequenced	Assisted reproduction’s Technologies	It was found that the plenty of Lactobacillus was favorable for positive results. In addition plenty of *Lactobacillus crispatus* and *iners*, respectively increased and decreased in the group favorable in comparison to unfavorable group.
**Patel *et al*., 2022**	31 women (11 fertile and 20 infertile, being 10 with failure appellant and 10 with infertility unexplained)	Does not portray	The samples faecal and vaginal, the regions variables V2-V3 of the gene 16S rRNA were amplified and sequenced.	Fertilization In vitro (IVF) and Injection intracytoplasmic the of sperm (ICSI)	The vaginal microbiota was dominated by genre *Lactobacillus*, with *Lactobacillus iners* being the most species abundant among the groups. Compared with the infertile cohort, the growth excessive of bacteria anaerobic, associated with dysbiosis vaginal, like *Leptotrichia* and *Snethia*, occurred in the controls.

### The composition of the vaginal microbiome of fertile and infertile women

The studies compare the vaginal microbiome of fertile and infertile women. Since 512 (25.45%) of fertile women showed dominance of *Lactobacillus* in the vaginal microbiota, while 1,499 (74.54%) infertile women had a higher microbial diversity and decrease in the number of *Lactobacillus*.

*Gardnerella spp*. was present in the microbiome of infertile women in 66.6%, followed by *Atopobium spp*. and *Prevotella spp*. in 38.8%, *Escherichia coli* in 27.7%, *Streptococcus spp., Sneathia* and *Staphylococcus* in 22.2%, *Enterococcus spp*. by 16.6%. Other microorganisms cited in infertile women were: *Candida spp*. (Babu *et al*., 2017), *Ureaplasma spp*. (Wee *et al*., 2018), *Chlamydia trachomatis* (Graspeuntner *et al*., 2018), *Mycoplasma hominis* (Sezer *et al*., 2022).

Some studies have associated the presence of *Lactobacillus crispatus* as an important predictor of pregnancy (Graspeuntner *et al*., 2018; Bernabeu *et al*., 2019; Haahr *et al*., 2019; Koedooder *et al*., 2019; Villani *et al*., 2022). On the other hand, studies of Graspeuntner *et al*. (2018), Haahr *et al*. (2019) and Villani *et al*. (2022) associated the presence of *Lactobacillus iners* with a more varied microbiota.

The studies also address changes in the vaginal microbiome during use of assisted reproduction techniques. Carosso *et al*. (2020) noted that despite the dominance of *Lactobacillus* in the vaginal microbiome is permanent after the IVF cycle, there was a decrease in abundance. The same was seen by Koedooder *et al*. (2021) and Villani *et al*. (2022).

## DISCUSSION

In 25.45% of the articles analyzed in the present study, it was observed that the microbiota of fertile women is constituted by a dominance of *Lactobacillus spp. Lactobacillus spp*. were first described in 1901, being a genus of aerobic bacteria in the form of rod and immobile, Gram-positive, non-spore-forming, acid-tolerant and capable of produce lactic acid by fermentation of carbohydrates from the phylum *Firmicutes* (Chee *et al*., 2020; Dempsey & Corr, 2022; Zhang *et al*., 2019).

Members of the *Lactobacillus* genus are abundant and predominant in the vaginal niche of healthy women of reproductive age, reaching a concentration of 10 7 cfu/mL of sample vaginal and 80% of all microbial content. Including: *Lactobacillus crispatus, Lactobacillus gasseri, Lactobacillus iners* and *Lactobacillus jensenii* (Chee *et al*., 2020; Parolin *et al*., 2021).

It is widely demonstrated that vaginal lactobacilli are involved in maintenance of the state of vaginal eubiosis and one of the main functions of lactobacilli is to activate the glycogen metabolism. Glycogen produced by vaginal epithelial cells is transformed into lactic acid, inducing a low vaginal pH (3.8-4.4). This creates an environment unfavorable for the growth of pathogenic bacteria and sexually transmitted infections (Di Simone *et al*., 2020; Parolin *et al.*, 2021).

In the study by Koedooder *et al*. (2019) pay attention to the dominance of *Lactobacillus crispatus* and reported to be an important factor in predicting pregnancy (<60%). In literature we found that *L. crispatus* produces lactic acid and other compounds that are potent inhibitors of associated bacterial species, mainly bacterial vaginosis. Therefore, *L. crispatus* seems to be a promising species because it is associated with vaginal health and negatively associated with bacterial vaginosis and preterm birth (Abdelmaksoud *et al*., 2016).

Infertile women (74.54%) had a greater diversity of microorganisms and a decrease in the proportion of *Lactobacillus* in the vaginal microbiota when compared to fertile women. The literature has portrayed that alterations in the dominance of lactobacilli and a microbiota with high bacterial diversity, are associated with an increased risk of infections, spontaneous preterm birth, and pelvic inflammatory disease (Di Simone *et al*., 2020).

In the study by Babu *et al.* (2017), it was observed that women with infertility had a low percentage of *Lactobacillus* and had vaginosis asymptomatic. In the literature we found that women with bacterial vaginosis, the microbiome of lactobacilli, which produce hydrogen peroxide, are responsible for maintaining from an acidic environment, which ends up being replaced by invasive pathogens, such as *Gardnerella vaginalis, Prevotella spp*. and *Mobiluncus spp*.. This substitution promotes a pH that sets the environment for bacterial vaginosis, in addition, *G. vaginalis* produces a biofilm that provides a matrix for the adhesion of other pathogenic bacteria, in addition to hinder the penetration of antibiotic therapy and eradication of the infection (Bagnall & Rizzolo, 2017).

In 66.6% of the articles analyzed in the present study, the presence of *Gardnerella spp*. in the vaginal microbiome of infertile women. In 1955, *Gardnerella* was known as the main organism involved in bacterial vaginosis, being a Gram-facultative anaerobic variable, its infection results in higher vaginal pH, thin discharge, fishy odor and presence of epithelial cells covered with bacteria. Sometimes the infection can is asymptomatic, even so it can be accompanied by serious consequences for the health conditions, such as premature birth and pelvic inflammatory disease, and may facilitate the acquisition of sexually transmitted infections (Morrill *et al*., 2020; Wong *et al*., 2018).

Bacterial vaginosis is a dysbiosis, as it causes a condition in which there is a decrease in of lactobacilli levels and overgrowth of several bacteria from other taxonomic groups (*Gardnerella, Atopobium, Mobiluncus, Prevotella, Bacteroides, Anaerococcus, Peptostreptococcus, Sneathia, Leptotrichia* and members of the *Clostridia* class, among others). Proposes that vaginal dysbiosis is linked to inflammatory states and is associated with adverse obstetrics. Bacterial vaginosis has been linked to infertility, although the cause that leads patients to be infertile has not yet been elucidated, it is known that the association between microbiota of a patient with bacterial vaginosis and subsequent inflammation can lead to reduced fertility (Di Simone *et al*., 2020; Morrill *et al*., 2020; Reiter & Kellogg Spadt, 2019).

It was observed in 38.8% of the articles analyzed in the present study the presence of microorganism *Atopobium spp*. in the microbiota of infertile women. *Atopobium spp*. was described in 1999, has a variable morphology from elongated cocci to bacilli, with Grampositive, and may be present singly, in pairs or in small chains. although already its presence in the microbiota of healthy women has been verified, it has been demonstrated that *Atopobium* is more frequently found in the vaginal microbiota of patients with bacterial vaginosis, as it is an important component in the formation of biofilms (Rodriguez Jovita *et al*., 1999; Zhou *et al*., 2004).

The microorganism *Prevotella spp*. was also found in the microbiota of infertile women in 38.8% of the analyzed articles. *Prevotella spp*. was named after the French microbiologist A.R. Prevot, a pioneer in anaerobic microbiology, is a Gram-anaerobic negative, which stains weakly by Gram, of the phylum *Bacteroidetes*, which also includes the clinically important genera *Bacteroides* and *Porphyromonas*. Are classically considered commensal bacteria due to their extensive presence in the healthy human body and its rare involvement in infections. Only a few strains have been reported to give rise to endogenous opportunistic infections, including chronic infections, abscesses and anaerobic pneumonia. However, it has been associated that the interaction between *Prevotella* and the immune system, can promote inflammatory disease and its abundance has been seen increases with the severity of bacterial vaginosis, in addition to being inversely correlated with the presence of *Lactobacillus* (Larsen, 2017; Murray *et al*., 2020).

As for assisted reproduction techniques, it was observed that patients undergoing IVF or ICSI showed a decrease in *Lactobacillus* levels and an increase in bacteria such as: *Staphylococcus, Atopobium, Escherichia-Shigella* and *Prevotella*. The studies also pointed out that women with recurrent implantation failures had a greater microbial diversity, in addition to showing a decrease in the number of *Lactobacillus*, reporting that this decrease plays an important role in the pathogenesis of recurring deployment. The literature points out that genital dysbiosis (for example, vaginal or endometrial tissue) was associated with lower odds of live births in reproductive Technologies (ART), by decreasing pregnancy rates and increasing the risk of miscarriages (Mauries *et al*., 2021).

Moreno *et al*. (2016) report that the existence of an endometrial microbiota highly stable during the acquisition of endometrial receptivity is a predictive factor positive for successful implementation. However, the pathological modification of your profile is associated with poor reproductive outcomes for in vitro fertilization (IVF) patients. He was demonstrated that the presence of a microbiota not dominated by *Lactobacillus* in a receptive endometrium was associated with significant decreases at implantation (60.7% *vs*. 23.1%; *P*=0.02).

Moreno *et al*. (2022) analyzed the endometrial microbiome of 342 infertile women. clinics in Europe, America and Asia. In their results, they observed that women with presence of microorganisms such as *Atopobium, Bifidobacterium, Chryseobacterium, Gardnerella, Haemophilus, Klebsiella, Neisseria, Staphylococcus* and *Streptococcus* do not were successful in the in vitro fertilization (IVF) technique, but women whose microbiome showed dominance of *Lactobacillus* were successful in the procedure. Therefore, the analysis of the composition of the endometrial microbiota before the transfer of the embryo is a useful biomarker for predicting reproductive outcome, offering an opportunity to further improve diagnostic and treatment strategies.

Currently, there are genomic diagnostic tools for the receptivity endometrial tissue based on transcriptomic signature, composed of a microarray and a bioinformatic predictor for endometrial dating and to detect pathology of endometrial origin, the ERA - Endometrial Receptivity ARRAY. Díaz-Gimeno *et al*. (2011) performed a clinical trial with healthy women (88) with implantation failure (5) or hydrosalpinx (2) and exposed the ERA with a diagnostic tool that can be used clinically in reproductive medicine and gynecology to assess receptivity endometrial. Garrido-Gómez *et al*. (2013) corroborates by pointing out the possibility of ERA taking a new clinical concept of personalized embryo transfer by verifying the optimal day of endometrial receptivity, identified individually on a case-by-case basis.

It is important to report that there are tests that perform the metagenomic analysis of the endometrial microbiome to allow a better reproductive prognosis. A endometrial biopsy, which provides proportion of healthy bacteria, including *Lactobacillus spp*., in addition to classifying as normal, abnormal and dysbiotic microbiota or very low. These tests are based on Next Generation Sequencing (NGS) technology. to provide information on the endometrial microbiome, based on the DNA extraction and 16S ribosomal RNA gene sequencing from bacteria. Therefore, provides a microbiological view of the endometrium with the aim of improving management patients’ clinic.

A diagnostic method that also shows promising results is the real-time polymerase chain (RT-PCR) that can identify bacterial DNA with 75% sensitivity and 100% specificity, allowing the identification of bacteria cultivable or not, even without signs of infection (Borges Júnior *et al*., 2020).

It is known that a limiting factor for the use of such methodologies is need expensive machinery, inputs that are sometimes lacking in the market and of specialized work, since the professionals to conduct certain technologies of diagnosis require a high degree of specialization, with well-in-depth knowledge of molecular diagnostics and bioinformatics analysis, being an important impact factor on the quality of reactions that are introduced and offered to customers.

Today such tests are used in patients who had implantation failure, However, since the probability of conception rate is around 25%-30% in the face of a cycle and that 75% of implantation failures do not have clinical knowledge (Borges Júnior *et al*., 2020). It is suggested that such methodologies should be used as a factor to prevent all women who will be assisted by reproductive techniques.

## CONCLUSION

The vaginal microbiome plays an important role in reproductive health. Therefore, analyzing bacterial patterns would allow a personalized diagnosis based on microbiota, which could favor personalized therapy for the prevention and treatment of certain diseases. We observed that the vaginal microbiome of fertile women showed dominance of *Lactobacillus*, while infertile women showed a decrease in *Lactobacillus* and increase in the variety of microorganisms. It was also seen that the *Lactobacillus* dominance is associated with positive predictive outcomes in reproduction and that vaginal dysbiosis is associated with unfavorable outcomes. As far as we know in Brazil it is not necessary to evaluate the vaginal microbiome for the use of assisted reproduction. But, we suggest that perhaps assessing the vaginal microenvironment would be a approach of interest, mainly for a favorable embryo implantation and a positive pregnancy outcome. Currently, diagnostic tools capable of to identify pathogenic bacteria in the female reproductive tract, these tests should be used in order to provide an opportunity to improve the clinical management of infertile patients.

## References

[r1] Abdelmaksoud AA, Koparde VN, Sheth NU, Serrano MG, Glascock AL, Fettweis JM, Strauss JF, Buck GA, Jefferson KK. (2016). Comparison of Lactobacillus crispatus isolates from
Lactobacillus-dominated vaginal microbiomes with isolates from microbiomes
containing bacterial vaginosis-associated bacteria. Microbiology (Reading).

[r2] Auriemma RS, Scairati R, Del Vecchio G, Liccardi A, Verde N, Pirchio R, Pivonello R, Ercolini D, Colao A. (2021). The Vaginal Microbiome: A Long Urogenital Colonization Throughout
Woman Life. Front Cell Infect Microbiol.

[r3] Azpiroz MA, Orguilia L, Palacio MI, Malpartida A, Mayol S, Mor G, Gutiérrez G. (2021). Potential biomarkers of infertility associated with microbiome
imbalances. Am J Reprod Immunol.

[r4] (2017). Flora and Incidence of Asymptomatic Vaginosis among Healthy Women
and in Women with Infertility Problems of Reproductive Age. J Clin Diagn Res.

[r5] Bagnall P, Rizzolo D. (2017). Bacterial vaginosis: A practical review. JAAPA.

[r6] Bernabeu A, Lledo B, Díaz MC, Lozano FM, Ruiz V, Fuentes A, Lopez-Pineda A, Moliner B, Castillo JC, Ortiz JA, Ten J, Llacer J, Carratala-Munuera C, Orozco-Beltran D, Quesada JA, Bernabeu R. (2019). Effect of the vaginal microbiome on the pregnancy rate in women
receiving assisted reproductive treatment. J Assist Reprod Genet.

[r7] Borges Júnior E, Braga DPAF, Setti AS. (2020). Reprodução Humana Assistida - Associação
Instituto Sapientiae.

[r8] Buchta V. (2018). Vaginal microbiome. Ceska Gynekol.

[r9] Carosso A, Revelli A, Gennarelli G, Canosa S, Cosma S, Borella F, Tancredi A, Paschero C, Boatti L, Zanotto E, Sidoti F, Bottino P, Costa C, Cavallo R, Benedetto C. (2020). Controlled ovarian stimulation and progesterone supplementation
affect vaginal and endometrial microbiota in IVF cycles: a pilot
study. J Assist Reprod Genet.

[r10] Ceccarani C, Foschi C, Parolin C, D’Antuono A, Gaspari V, Consolandi C, Laghi L, Camboni T, Vitali B, Severgnini M, Marangoni A. (2019). Diversity of vaginal microbiome and metabolome during genital
infections. Sci Rep.

[r11] Chee WJY, Chew SY, Than LTL. (2020). Vaginal microbiota and the potential of Lactobacillus derivatives
in maintaining vaginal health. Microb Cell Fact.

[r12] Dempsey E, Corr SC. (2022). Lactobacillus spp. for Gastrointestinal Health: Current and
Future Perspectives. Front Immunol.

[r13] Di Simone N, Santamaria Ortiz A, Specchia M, Tersigni C, Villa P, Gasbarrini A, Scambia G, D’Ippolito S. (2020). Recent Insights on the Maternal Microbiota: Impact on Pregnancy
Outcomes. Front Immunol.

[r14] Díaz-Gimeno P, Horcajadas JA, Martínez-Conejero JA, Esteban FJ, Alamá P, Pellicer A, Simón C. (2011). A genomic diagnostic tool for human endometrial receptivity based
on the transcriptomic signature. Fertil Steril.

[r15] Esteves SC, Humaidan P, Roque M, Agarwal A. (2019). Female infertility and assisted reproductive
technology. Panminerva Med.

[r16] Félis KC, Campos AA, Silva AM, Carvalho IG, Pargeon JP, Almeida RJ. (2019). Repercussões psicossociais da infertilidade inexplicada em
mulheres. Nursing (São Paulo).

[r17] Foizer BRR, Silva KR, Vieira JDG, Amaral WN. (2014). Microbiological contamination in the laboratory of human
reproduction and its implications for the success of assisted
reproduction. Reprod Clim.

[r18] Franasiak JM, Scott RT Jr. (2015). Reproductive tract microbiome in assisted reproductive
technologies. Fertil Steril.

[r19] Fu M, Zhang X, Liang Y, Lin S, Qian W, Fan S. (2020). Alterations in Vaginal Microbiota and Associated Metabolome in
Women with Recurrent Implantation Failure. mBio.

[r20] Garrido-Gómez T, Ruiz-Alonso M, Blesa D, Diaz-Gimeno P, Vilella F, Simón C. (2013). Profiling the gene signature of endometrial receptivity: clinical
results. Fertil Steril.

[r21] Godha K, Tucker KM, Biehl C, Archer DF, Mirkin S. (2018). Human vaginal pH and microbiota: an update. Gynecol Endocrinol.

[r22] Graspeuntner S, Bohlmann MK, Gillmann K, Speer R, Kuenzel S, Mark H, Hoellen F, Lettau R, Griesinger G, König IR, Baines JF, Rupp J. (2018). Microbiota-based analysis reveals specific bacterial traits and a
novel strategy for the diagnosis of infectious infertility. PLoS One.

[r23] Haahr T, Humaidan P, Elbaek HO, Alsbjerg B, Laursen RJ, Rygaard K, Johannesen TB, Andersen PS, Ng KL, Jensen JS. (2019). Vaginal Microbiota and In Vitro Fertilization Outcomes:
Development of a Simple Diagnostic Tool to Predict Patients at Risk of a
Poor Reproductive Outcome. J Infect Dis.

[r24] Hao X, Li P, Wu S, Tan J. (2021). Association of the Cervical Microbiota With Pregnancy Outcome in
a Subfertile Population Undergoing In Vitro Fertilization: A Case-Control
Study. Front Cell Infect Microbiol.

[r25] Hong X, Ma J, Yin J, Fang S, Geng J, Zhao H, Zhu M, Ye M, Zhu X, Xuan Y, Wang B. (2020). The association between vaginal microbiota and female
infertility: a systematic review and meta-analysis. Arch Gynecol Obstet.

[r26] Jespers V, Menten J, Smet H, Poradosú S, Abdellati S, Verhelst R, Hardy L, Buvé A, Crucitti T. (2012). Quantification of bacterial species of the vaginal microbiome in
different groups of women, using nucleic acid amplification
tests. BMC Microbiol.

[r27] Karaer A, Doğan B, Günal S, Tuncay G, Arda Düz S, Ünver T, Tecellioğlu N. (2021). The vaginal microbiota composition of women undergoing assisted
reproduction: a prospective cohort study. BJOG.

[r28] Koedooder R, Singer M, Schoenmakers S, Savelkoul PHM, Morré SA, de Jonge JD, Poort L, Cuypers WJSS, Beckers NGM, Broekmans FJM, Cohlen BJ, den Hartog JE, Fleischer K, Lambalk CB, Smeenk JMJS, Budding AE, Laven JSE. (2019). The vaginal microbiome as a predictor for outcome of in vitro
fertilization with or without intracytoplasmic sperm injection: a
prospective study. Hum Reprod.

[r29] Koedooder R, Maghdid DM, Beckers NGM, Schoenmakers S, Kok DJ, Laven JSE. (2021). Dynamics of the urinary microbiome in pregnancy and the
coincidental predictive value of the microbiota for IVF/IVF-ICSI
outcome. Reprod Biomed Online.

[r30] Larsen JM. (2017). The immune response to Prevotella bacteria in chronic
inflammatory disease. Immunology.

[r31] Lüll K, Saare M, Peters M, Kakhiani E, Zhdanova A, Salumets A, Boyarsky K, Org E. (2022). Differences in microbial profile of endometrial fluid and tissue
samples in women with in vitro fertilization failure are driven by
Lactobacillus abundance. Acta Obstet Gynecol Scand.

[r32] Martin DH. (2012). The microbiota of the vagina and its influence on women’s health
and disease. Am J Med Sci.

[r33] Mauries C, Ranisavljevic N, Gallet R, Fournier A, Gala A, Ferrières-Hoa A, Brouillet S, Hamamah S. (2021). Assessment of genital microbiota: An emerging approach in
assisted reproductive techniques. Gynecol Obstet Fertil Senol.

[r34] Mendling W., Schwiertz A (2016). Microbiota of the Human Body. Implications in Health and
Disease.

[r35] Moreno I, Codoñer FM, Vilella F, Valbuena D, Martinez-Blanch JF, Jimenez-Almazán J, Alonso R, Alamá P, Remohí J, Pellicer A, Ramon D, Simon C. (2016). Evidence that the endometrial microbiota has an effect on
implantation success or failure. Am J Obstet Gynecol.

[r36] Moreno I, Garcia-Grau I, Perez-Villaroya D, Gonzalez-Monfort M, Bahçeci M, Barrionuevo MJ, Taguchi S, Puente E, Dimattina M, Lim MW, Meneghini G, Aubuchon M, Leondires M, Izquierdo A, Perez-Olgiati M, Chavez A, Seethram K, Bau D, Gomez C, Valbuena D (2022). Endometrial microbiota composition is associated with
reproductive outcome in infertile patients. Microbiome.

[r37] Morrill S, Gilbert NM, Lewis AL. (2020). Gardnerella vaginalis as a Cause of Bacterial Vaginosis:
Appraisal of the Evidence From in vivo Models. Front Cell Infect Microbiol.

[r38] Murray PR, Rosenthal KS, Pfaller MA. (2020). Medical microbiology.

[r39] Oliveira MS, Medeiros FC, Eleutério Junior J. (2019). Infertility and the vaginal microbiome: review
study. J Bras Doenças Sex Transm.

[r40] Parolin C, Croatti V, Laghi L, Giordani B, Tondi MR, De Gregorio PR, Foschi C, Vitali B. (2021). Lactobacillus Biofilms Influence Anti-Candida
Activity. Front Microbiol.

[r41] Patel N, Patel N, Pal S, Nathani N, Pandit R, Patel M, Patel N, Joshi C, Parekh B. (2022). Distinct gut and vaginal microbiota profile in women with
recurrent implantation failure and unexplained infertility. BMC Womens Health.

[r42] Reiter S, Kellogg Spadt S. (2019). Bacterial vaginosis: a primer for clinicians. Postgrad Med.

[r43] Requena T, Velasco M. (2021). The human microbiome in sickness and in health. Rev Clin Esp.

[r44] Rodriguez Jovita M, Collins MD, Sjödén B, Falsen E. (1999). Characterization of a novel Atopobium isolate from the human
vagina: description of Atopobium vaginae sp. nov. Int J Syst Bacteriol.

[r45] Sezer O, Soyer Çalışkan C, Celik S, Kilic SS, Kuruoglu T, Unluguzel Ustun G, Yurtcu N. (2022). Assessment of vaginal and endometrial microbiota by real-time PCR
in women with unexplained infertility. J Obstet Gynaecol Res.

[r46] Sirota I, Zarek SM, Segars JH. (2014). Potential influence of the microbiome on infertility and assisted
reproductive technology. Semin Reprod Med.

[r47] Souza KKPC, Alves OF. (2016). As principais técnicas de reprodução humana
assistida. Saúde Ciênc Ação.

[r48] Starc A, Trampuš M, Pavan Jukić D, Rotim C, Jukić T, Polona Mivšek A. (2019). Infertility and sexual dysfunctions: a systematic literature
review. Acta Clin Croat.

[r49] Sun N, Ding H, Yu H, Ji Y, Xifang X, Pang W, Wang X, Zhang Q, Li W. (2021). Comprehensive Characterization of Microbial Community in the
Female Genital Tract of Reproductive-Aged Women in China. Front Cell Infect Microbiol.

[r50] van Oostrum N, De Sutter P, Meys J, Verstraelen H. (2013). Risks associated with bacterial vaginosis in infertility
patients: a systematic review and meta-analysis. Hum Reprod.

[r51] Villani A, Fontana A, Barone S, de Stefani S, Primiterra M, Copetti M, Panebianco C, Parri C, Sciannamè N, Quitadamo PA, Tiezzi A, Santana L, Maglione A, D’Amato F, Perri F, Palini S, Pazienza V. (2022). Identifying Predictive Bacterial Markers from Cervical Swab
Microbiota on Pregnancy Outcome in Woman Undergoing Assisted Reproductive
Technologies. J Clin Med.

[r52] Wee BA, Thomas M, Sweeney EL, Frentiu FD, Samios M, Ravel J, Gajer P, Myers G, Timms P, Allan JA, Huston WM. (2018). A retrospective pilot study to determine whether the reproductive
tract microbiota differs between women with a history of infertility and
fertile women. Aust N Z J Obstet Gynaecol.

[r53] Weiss GA, Hennet T. (2017). Mechanisms and consequences of intestinal
dysbiosis. Cell Mol Life Sci.

[r54] Wong YP, Tan GC, Wong KK, Anushia S, Cheah FC. (2018). Gardnerella vaginalis in perinatology: An overview of the
clinicopathological correlation. Malays J Pathol.

[r55] World Health Organization (WHO) (2020). Infertility.

[r56] Younes JA, Lievens E, Hummelen R, van der Westen R, Reid G, Petrova MI. (2018). Women and Their Microbes: The Unexpected
Friendship. Trends Microbiol.

[r57] Zegers-Hochschild F, Crosby JA, Musri C, Souza MDCB, Martinez AG, Silva AA, Mojarra JM, Masoli D, Posada N. (2020). Assisted reproductive techniques in Latin America: The Latin
American Registry, 2017. JBRA Assist Reprod.

[r58] Zhang Z, Hou Q, Wang Y, Li W, Zhao H, Sun Z, Guo Z. (2019). Lactobacillus zhachilii sp. nov., a lactic acid bacterium
isolated from Zha-Chili. Int J Syst Evol Microbiol.

[r59] Zhao C, Wei Z, Yang J, Zhang J, Yu C, Yang A, Zhang M, Zhang L, Wang Y, Mu X, Heng X, Yang H, Gai Z, Wang X, Zhang L. (2020). Characterization of the Vaginal Microbiome in Women with
Infertility and Its Potential Correlation with Hormone Stimulation during In
Vitro Fertilization Surgery. mSystems.

[r60] Zhou X, Bent SJ, Schneider MG, Davis CC, Islam MR, Forney LJ. (2004). Characterization of vaginal microbial communities in adult
healthy women using cultivation-independent methods. Microbiology (Reading).

